# Children with special health care needs attending emergency department in Italy: analysis of 3479 cases

**DOI:** 10.1186/s13052-020-00937-x

**Published:** 2020-11-23

**Authors:** Paola Cianci, Valeria D’Apolito, Alex Moretti, Massimo Barbagallo, Sabrina Paci, Maria Teresa Carbone, Riccardo Lubrano, Antonio Urbino, Carlo Dionisi Vici, Luigi Memo, Giuseppe Zampino, Giancarlo La Marca, Alberto Villani, Giovanni Corsello, Angelo Selicorni, on behalf of the Italian Society of Pediatrics (SIP), the Italian Society of Pediatric Genetic Diseases and Congenital Disabilities (SIMGePed) the Italian Society of Pediatric Emergency Medicine (SIMEUP), the Italian Society For The Study Of Inborn Metabolic Disorders And Newborn Screening (SIMMENS) and Members of Italian Network, A. Campania, C. Geremia, A. Urbino, E. Castagno, S. Masi, G. Poggi, M. Vestri, E. Fossali, A. Rocchi, L. Da Dalt, A. Arrighini, S. Chiappa, S. Renna, E. Piccotti, C. Borgna, M. R. Govoni, A. Biondi, C. Fossati, L. Iughetti, P. Bertolani, A. Salvatoni, M. Agosti, F. Fucà, A. Ilardi, S. Giuffrida, V. Di Guardo, L. Memo, S. Boni, L. D’Antiga, M. Ruggeri, A. Chiaretti, G. Zampino, S. Amarri, A. Peduto, F. Bernardi, I. Corsini, G. L. De Angelis, C. Ruberto, G. V. Zuccotti, C. Stringhi, G. Lombardi, C. Salladini, S. Di Michele, L. Parola, A. Porta, G. Biasucci, M. Bellini, M. T. Ortisi, E. Apuril, F. Midulla, L. Tarani, G. Parlapiano, D. Lietti, C. Sforzini, G. L. Marseglia, S. Savasta, R. Falsaperla, M. C. Vitaliti, F. Chiarelli, N. Rossi, G. Banderali, R. Giacchero, L. Bernardo, F. Pinto, E. Fabiani, A. Ficcadenti, G. Pellegrini, S. Giacoma, P. Biban, S. Spada, V. Tipo, M. T. Carbone, C. Ghitti, S. Bolognini, G. Mariani, A. Russo, M. G. Colella, A. Verrico, P. Bruni, D. Poddighe, G. Cagnoli, F. Morandi, A. Gadaleta, E. Barbi, I. I Bruno, R. Graziano, P. Sgaramella, M. P. Catalani, I. Baldoni, G. Colarusso, G. Galvagno, A. P. Barone, A. Longo, G. Nardella, G. Portale, G. Garigali, G. Bona, M. Erbela, R. Agostiniani, L. Nanni, E. Schieven, M. Donà, T. Varisco, F. Russo, V. A. Di Stefano, F. Di Pietro, L. Tarallo, L. Imperato, G. Parisi, R. Salzano, G. Raiola, V. Talarico, R. Bellù, A. Cannone, P. Ferrante

**Affiliations:** 1 Department of Pediatrics, ASST-Lariana, Hospital “Sant’Anna”, Como, Italy; 2 Woman and Child Department, Hospital “F. Del Ponte”, University of Insubria, Varese, Italy; 3 Clinical Pediatric Genetics Unit, MBBM Foundation, S. Gerardo Hospital, Monza, Italy; 4 Pediatric Unit, Azienda di rilievo nazionale ARNAS “Garibaldi”, Catania, Italy; 5 Department of Pediatrics, San Paolo Hospital, Milan, Italy; 6 Screening Center Fenilchetonuria, SS. Annunziata Hospital, ASL Na1, Naples, Italy; 7 Department of Pediatrics, La Sapienza University of Rome- Hospital of Latina, Rome, Italy; 8 Emergency Department, Ospedale Infantile Regina Margherita, Torino, Italy; 9 Metabolic Diseases Unit, Bambino Gesù Children Hospital and Research Institute, Rome, Italy; 10 Pediatric Unit, San Martino Hospital, Belluno, Italy; 11 Center for Rare Disease and Congenital Defects, Fondazione Policlinico Universitario A. Gemelli, Catholic University, Rome, Italy; 12 Department of Experimental and Clinical Biomedical Sciences, University of Florence and Head, Newborn Screening, Clinical Chemistry and Pharmacology Laboratory, Meyer Children’s Hospital, Florence, Italy; 13 Pediatric and Infectious Disease Unit, Bambino Gesu’ Children’s Hospital, IRCCS, Rome, Italy; 14 Clinical Pediatric and Neonatology Unit, Policlinic Hospital, Woman and Child Department, Palermo, Italy

**Keywords:** Children with special health care needs, Emergency department, Hospitalization rate, Syndromic disorders, Metabolic diseases, Neuromuscular diseases, Congenital skeletal condition, True isolated microcephaly, Isolated CNS malformation, Multiple AED therapy

## Abstract

**Background:**

Although children with special health care needs (CSHCN) represent a minority of the population, they go through more hospitalizations, more admissions to the Emergency Department (ED), and receive a major number of medical prescriptions, in comparison to general pediatric population. Objectives of the study were to determine the reasons for admission to the ED in Italian CSHCN, and to describe the association between patient’s demographic data, clinical history, and health services requirements.

**Methods:**

Ad hoc web site was created to collect retrospective data of 3479 visits of CSHCN to the ED in 58 Italian Hospitals.

**Results:**

Seventy-two percent of patients admitted to ED were affected by a previously defined medical condition. Most of the ED admissions were children with syndromic conditions (54%). 44.2% of the ED admissions were registered during the night-time and/or at the weekends. The hospitalization rate was of 45.6% among patients admitted to the ED. The most common reason for admission to the ED was the presence of respiratory symptoms (26.6%), followed by gastrointestinal problems (21.3%) and neurological disorders (18.2%). 51.4% of the access were classified as ‘urgent’, with a red/yellow triage code. Considering the type of ED, 61.9% of the visits were conducted at the Pediatric EDs (PedEDs), 33.5% at the Functional EDs (FunEDs) and 4.6% at the Dedicated EDs (DedEDs). Patients with more complex clinical presentation were more likely to be evaluated at the PedEDs. CSHCN underwent to a higher number of medical procedures at the PedEDs, more in comparison to other EDs. Children with medical devices were directed to a PedED quite exclusively when in need for medical attention. Subjects under multiple anti-epileptic drug therapy attended to PedEDs or FunEDs generally. Patients affected by metabolic diseases were more likely to look for medical attention at FunEDs. Syndromic patients mostly required medical attention at the DedEDs.

**Conclusions:**

Access of CSHCN to an ED is not infrequent. For this reason, it is fundamental for pediatricians working in any kind of ED to increase their general knowledge about CHSCN and to gain expertise in the management of such patients and their related medical complexity.

## Introduction

The Federal Maternal and Child Health Bureau defines children with special health care needs (CSHCN) as “those who have or are at increased risk for a chronic physical, developmental, behavioral, or emotional condition and require health care and related services of a type or amount beyond that required by children generally” [1]. Although 13 to 18% of the children are considered to have special needs, there is a consistent discrepancy in terms of medical complexity, functional limitations, and resource needs among CSHCN [2]. Research shows that CSHCN are a heterogeneous group of patients whose health problems manifest throughout time with increasing complexity levels, functional limitations, comorbidities and need for specific health services [3]. These patients are commonly identified as children who require health services above the average, and these are expected to be required for more than 12 months [4], sometimes depending on assistive devices for daily living [5]. The group of CSHCN patients is extremely wide and includes several conditions such as syndromic patients, patients affected by metabolic diseases, patients with neurological problems, such as severe seizure or malformations of the central nervous system, patients with congenital skeletal conditions and medical devices users.

Scientific Literature highlights that American CSHCN have health care expenses that are 3 times higher than general pediatric population. CSHCN also have 4 times the number of hospitalizations, twice as many physician visits, 1.5 times as many Emergency Department (ED) visits, and receive 5 times the number of prescriptions [6]. Furthermore, in the United States (US) CSHCN have a related risk of 3.3 for Intensive Care Unit (ICU) admission due to their chronic condition, compared with previously healthy individuals. 32% of ICU admissions were potentially preventable [7].

Due to the complexity of CSHCN, it might be a challenge for physicians to distinguish whether the symptoms are associated to the chronic condition or not. Several studies addressed the need for training programs to improve emergency medical services in the assessment and treatment of CSCHN [8–10].

The aim of this study was to determine the reason of access to the ED of CSHCN, their clinical history, the ED management and outcome. Secondary aims were to describe timing, location and reason of access to the ED and to determine the required/received health care.

## Patients and methods

ED accesses CSHCN were retrospectively analyzed from December 1st 2015 to May 31st 2016 in 58 Italian hospitals using an ad hoc web site. Pediatricians from different EDs had to fill in a simple anonymous web form on CSHCN ED access. Location and type of ED were registered. Purely pediatric EDs (PedEDs) are the most specialized EDs for children in Italy, since children are taken care entirely by pediatricians and pediatric nurses. PedEDs represent a specific Department in pediatric Hospitals. Concerning functional pediatric EDs (FunEDs), the triage is performed by the general ED where children are referred to a specific pediatric area within the general ED. At the dedicated pediatric EDs (DedEDs), pediatricians are generally not included in the hospital’s ED team, therefore children are triaged and visited by a general ED physician who asks for pediatric consult only in case of need. Time of the arrival to the ED was defined and registered, distinguishing two-time sets: a) from 7:00 a.m. to 8:00 p.m. on weekdays and b) during the night-time from 8:00 p.m. to 7:00 a.m. or during the weekends.

Patients were assessed based on age (< 1 year, 1–2 years, 3–5 years, 6–11 years, 12–17 years, and > 18 years), sex and type of medical device used (central venous catheters, gastrostomy tubes, home non-invasive ventilation or oxygen therapy, cough assist equipment, tracheostomy).

The only inclusion criteria to access the study was to be affected by one of the below listed complex chronic conditions:

1. **Defined syndromic disorders**, diagnosed on the basis of clinical and molecular background
2. **Presumptive syndromic disorders,** suspected by the presence of multiple major malformations or the presence of one major malformation associated with dysmorphisms and/or intellectual delay.
3. **Defined inherited metabolic diseases**, diagnosed on the basis of biochemical and molecular background
4. **Presumptive inherited metabolic diseases,** suspected by the presence of metabolic abnormalities with/without specific histopathologic or ultrastructural abnormalities and/or dysmorphisms.
5. **Defined neuromuscular diseases**, diagnosed on the basis of clinical and molecular background
6. **Presumptive neuromuscular diseases**, suspected by the presence of electrophysiological, imaging or histological abnormalities.
7. **Defined constitutional bone disorders**, diagnosed on the basis of clinical, radiological and molecular background
8. **Presumptive constitutional bone disorders** suspected by the presence of skeletal abnormalities compatible with dysostoses or osteochondrodysplasias, based on clinical and radiological features.
9. **True isolated microcephaly**, defined as an occipitofrontal head circumference (OFC) ≤ − 3 standard deviation (SD) below the mean for sex, age, and ethnicity, not associated to other malformations or clinical problems, not included in any of the above listed categories [11].
10. **Isolated central nervous system (CSN) malformations,** not listed in any of the above categories
11. **Anti-epileptic drugs (AED) resistant patients with nonlesional epilepsy.** Children receiving more than one antiepileptic drug that was not classified in any of the above categories [12].
12. **Medical device users** such as cerebrospinal fluid (CSF) shunts, venous catheters, gastrostomy tubes, nasogastric tube, urologic catheters, home non-invasive ventilation or oxygen therapy and/or tracheostomy, not classified in any of the above categories.

Oncological patients, including users of medical devices, were excluded from the study if not belonging to any category of the eleven mentionedabove.

The reasons of access to the ED were classified according to patient’s main symptomatology. Access to the ED was categorized as one of the following: fever, pain, respiratory distress, gastrointestinal problems, cardiac disorders, neurological symptoms, hematological problems, osteoarticular disorders, device malfunction, trauma, metabolic alteration and ‘other symptoms’ whenever unable to be classified in any of the aforementioned categories.

In Italy, Triage Codes are classified in colors according to patient’s clinical condition. ‘Red code’ stands for a non-deferrable emergency, and a life-threatening condition, a ‘yellow code’ is urgent, a no immediate life-threatening condition, ‘green code’ is considered of low urgency and priority, with a deferrable medical care, and ‘white code’ which is non-urgent. Triage codes are established at the ED by specialized triage nurses [13]. The type of medical attention received by CSHCN were classified as follows:

- diagnostic procedures such as blood tests, instrumental exams or specialist consultations, required by the pediatrician at the ED based on the clinical evaluation.
- therapeutic procedures such as intravenous fluids, pharmacologic therapy or oxygen administration.
- cardiopulmonary resuscitation (CPR) and invasive procedures such as difficult intravenous access, pneumothorax drainage, central venous access, intraosseous access, paracentesis, lumbar puncture or thoracentesis.

Patient’s outcome as ‘admitted’ or ‘discharged’ was specified at the ED charts review.

The study population was analyzed using descriptive statistics. Variables were analyzed using Chi-square test and Kruskal-Wallis test. The level of statistical significance was set at *p* < 0.05. The statistical analysis was performed with the SPSS software program, version 26.

## Results

Fifty-eight Italian EDs were included in the study, 17/58 (29.3%) were PedEDs, 35/58 (60.3%) were FunEDs and 6/58 were DedEDs (10.3%). Data of 3479 ED visits were collected.

From the total ED visits, 1971/3479 (56.7%) were males and 1508/3479 (43.3%) were females. 370/3479 (10.6%) were aged less than 1 year, 773/3479 (22.2%) were between 1 and 2 years, 870/3479 (25%) were between 3 and 5 years, 884/3479 (25.4%) were between 6 and 11 years, 514/3479 (14.8%) were between 12 and 17 years and only 68/3479 (2%) were older than 18 years old. A great number of patients (783/3479, 22.5%) were users of medical devices, with an average of 1,3 devices per person (range 1–4) (Table 1).

**Table 1:** Clinical characteristics of patients

	N	%
**Total**	**3479**	**100**
**Gender**		
Male	1971	56.7
Female	1508	43.3
**Age**		
< 1 year	370	10.6
1–2 years	773	22.2
3–5 years	870	25.0
6–11 years	884	25.4
12–17 years	514	14.8
> 18 years	68	2.0
**Devices**		
Yes	783	22.5
No	2696	77.5
**Type of devices**		
Central venous catheters	147	14.1
Gastrostomy tubes (PEG or PEJ)	562	53.7
Home non invasive ventilation	75	7.2
Home oxigen therapy	49	4.7
Cough assist equipment	39	3.7
Tracheostomy	174	16.6
**Etiological diagnosis**		
**Defined diagnosis**	**2506**	**72.0**
Syndromic disorders	1358	54.2
Metabolic diseases	304	12.1
Neuromuscular diseases	139	5.5
Congenital skeletal condition	49	2.0
True isolated microcephaly	25	1.0
Isolated CNS malformations	194	7.7
Epilepsy under AED	437	17.4
**Presumptive diagnosis**	**973**	**28.0**
Syndromic disorders	533	54.8
Metabolic diseases	91	9.4
Neuromuscular diseases	54	5.5
Congenital skeletal condition	7	0.7
Medical devices users	288	29.6

*N* Number, *CSN* Central nervous system, *AED* Anti-epileptic drug, *PEG* Percutaneous endoscopic gastrostomy, *PEJ* Percutaneous endoscopic jejunostomy

Most of our patients, 2506/3479 (72%) had a defined etiological diagnosis. The majority of them, 1891/3479 (54.4%) were syndromic children (with a defined or presumptive diagnosis), followed by 437/3479 (13.6%) AED resistant nonlesional epilepsy patients, 395/3479 (11.4%) children with an inherited metabolic disease (with a defined or presumptive diagnosis), 193/3479 (5.5%) children with neuromuscular disease (with defined or presumptive diagnosis) and 288/3479 (8.3%) of the patients were listed as medical device users who did not classified as any of the prior categories (Table 1).

2155/3479 (61.9%) visits were conducted in PedEDs, 1164/3479 (33.5%) in FunEDs and only 160/3479 (4.6%) in DedEDs. Most of the admissions were between 7:00 a.m. to 8:00 p.m. on weekdays (1940/3479, 55.8%) while 1539/3479 (44.2%) were registered during the night-time (from 8:00 p.m. to 7:00 a.m.) and /or at the weekends.

Respiratory symptoms (926/3479, 26.6%) were the most common cause of admission, followed by gastrointestinal problems (742/3479, 21.3%) and neurological disorders (632/3479, 18.2%). Isolated fever represented 11.4% (398/3479) of the visits (Table 2).

**Table 2:** Demographic characteristics ED visits and

	N	%
**Reasons for ED access**		
Pain	187	5.4
Respiratory	926	26.6
Gastrointestinal	742	21.3
Cardiac	49	1.4
Neurological	632	18.2
Hematological	43	1.2
Osteoarticular	68	2.0
Device malfunctioning	136	3.9
Trauma	125	3.6
Metabolic failure	58	1.7
Fever	398	11.4
Other symptoms	115	3.3
**Colour-code triage**		
Red	142	4.1
Yellow	1647	47.3
Green	1588	45.6
White	102	2.9
**Specific procedures performed**		
Intravenous fluids	856	43.8
Pharmacologic therapy	586	30.0
Oxygen administration	423	21.6
CPR	41	2.1
Invasive procedures	49	2.5

*N* Number, *CPR* Cardiopulmonary resuscitation

1690/3479 access (48.6%) were classified as “non urgent” with white/green triage codes; the remaining 1789/3479 (51.4%) were classified as red/yellow triage codes (Table 2).

One thousand nine hundred fifty-five patients required therapeutic procedures, 856/1955 children (43.8%) required intravenous fluids, 586/1955 (30%) received pharmacologic therapy and 423/1955 (21.6%) oxygen administration. CPR was conducted in 41/1955 patients (2.1%). 49/1955 patients (2.5%) required invasive procedures, of which 43/49 (87.7%) had a difficult intravenous access, one patient had an intraosseous access placement (1/49; 2%) another patient had a paracentesis (1/49; 2%), two patients had a central venous access placement (2/49; 4%) and other two had a lumbar puncture (2/49; 4%) (Table 2).

Data were analyzed considering the type of ED. A statistical difference between the triage codes and the type of ED was evidenced. In fact, patients with an acute clinical condition (red/yellow codes) were more likely to be evaluated in PedEDs. On the contrary, patients with less severe codes (white/green) were directed to FunEDs or DedEDs in the same proportion (Table 3).

**Table 3:** Performances and outcome for ED visits

	PedED N (%)	FunED N (%)	DedED N (%)	Total N (%)	***P*** value ^a^
Total	2155	1164	160	3479	
**Colour-code triage**					
Red	109 (5.1)	29 (2.5)	4 (2.5)	142 (4.1)	0.001
Yellow	1275 (59.2)	327 (28.1)	45 (28.1)	1647 (47.3)	< 0.001
Green	749 (34.8)	738 (63.4)	101 (63.1)	1588 (45.6)	< 0.001
White	22 (1.0)	70 (6.0)	10 (6.3)	102 (2.9)	< 0.001
**ED arrival time**					
Day-time/weekday	1200 (55.7)	658 (56.5)	82 (51.2)	1940	0.449
Night-time/weekend	955 (44.4)	506 (43.4)	78 (48.8)	1539	
**Etiological diagnosis**					
Syndromic disorders	1125 (52.2)	652 (56.0)	114 (71.3)	1891	< 0.001
Inherited metabolic diseases	228 (10.6)	159 (13.7)	8 (5.0)	395	0.001
Neuromuscular diseases	118 (5.5)	57 (4.9)	18 (11.3)	193	0.004
Congenital skeletal condition	34 (1.6)	21 (1.8)	1 (0.6)	56	0.530
True microcephaly	14 (0.6)	9 (0.8)	2 (1.3)	25	0.662
Isolated CNS malformations	120 (5.6)	68 (5.8)	6 (3.8)	194	0.557
Epilepsy under AED	279 (12.9)	150 (2.9)	8 (5.0)	437	0.013
Medical devices users	237 (11.0)	48 (4.1)	3 (1.9)	288	< 0.001
**Reason for ED access**					
Pain	113 (5.2)	69 (5.9)	5 (3.1)	187	0.306
Respiratory	544 (25.2)	332 (28.5)	50 (31.3)	926	0.050
Gastrointestinal	475 (22.0)	239 (20.5)	28 (17.5)	742	0.288
Cardiac	37 (1.7)	12 (1.0)	0	49	0.084
Neurological	419 (19.4)	193 (16.6)	20 (12.5)	632	0.020
Hematological	28 (1.3)	14 (1.2)	1 (0.6)	43	0.752
Osteoarticular	42 (1.9)	16 (1.4)	10 (6.3)	68	< 0.001
Device malfunctioning	101 (4.7)	35 (3.0)	0	136	0.002
Trauma	64 (3.0)	42 (3.6)	19 (11.9)	125	< 0.001
Metabolic failure	37 (1.7)	17 (1.5)	4 (2.5)	58	0.603
Fever	247 (11.5)	130 (11.2)	21 (13.1)	398	0.766
Other symptoms	48 (2.2)	65 (5.6)	2 (1.3)	115	< 0.001
**Outcomes**					
Discharge	1073 (49.8)	700 (60.1)	116 (72.5)	1889 (54.3)	< 0.001
Hospitalization	1082 (50.2)	464 (39.9)	44 (27.5)	1590 (45.7)	
**Diagnostic procedures in DP**					
0	333 (31.0)	308 (44.0)	49 (42.2)	690/1889 (36.5)	< 0.001^b^
1	450 (41.9)	249 (35.6)	42 (36.2)	741/1889 (39.2)	
≥ 2	290 (27.0)	143 (20.4)	25 (21.6)	456/1889 (24.2)	
**Diagnostic procedures in HP**					
0	154 (14.2)	62 (13.4)	11 (25.0)	227/1590 (14.3)	0.008^b^
1	382 (35.3)	193 (41.6)	20 (45.5)	595/1590 (37.4)	
≥ 2	546 (50.5)	209 (45.0)	13 (29.5)	768/1590 (48.3)	

*N* Number, *DP* Discharged patients; *HP* Hospitalized patients, *AED* Anti-epilepsy drugs, *CSN* Central nervous system, *ED* Emergency department

^a^ Chi-squared test except where otherwise indicated ^b^ Kruskal-Wallis test

The day and time of access was similar in all types of EDs (*p* = 0,449) (Table 3).

Based on the etiology, medical device users preferred the PedEDs (*p* < 0,001), AED resistant nonlesional epilepsy patients (*p* = 0,013) mostly referred to PedEDs or FunEDs. Proportionally, a higher percentage of patients with metabolic diseases were directed to FunEDs (*p* = 0,001) even if the absolute majority of the visits were registered in the PedEDs due to the higher number of total visits in this type of ED. At the DedEDs, most CSHCN were syndromic (71.3%) (*p* < 0,001) and a less significant percentage of the patients were affected by a neuromuscular disease (*p* = 0,004) (Table 3).

Patients with acute neurological problems (*p* = 0,020), or a device malfunction (*p* = 0,002) were more likely to be assessed at PedEDs. Respiratory problems (*p* = 0,050) AND trauma (*p* < 0,001) were the two most frequent reasons of visit at DedEDs (Table 3).

Independently from the type of ED, a high proportion of subjects (2560/3479; 73.6%) required at least one diagnostic procedure at the first pediatric physical examination. CSHCN who were visited at PedEDs required a higher number (≥2) of diagnostic procedures before discharge (27.0%) in comparison to CSHCN that were visited in other types of EDs (20.4% at FunEDs and 21.6% at DedEDs, respectively). The correlation between the required number of diagnostic procedures and the type of ED was significant (*p* < 0,001) (Table 3).

Table 4 shows the amount of diagnostic procedures required according to the diagnosis. Children affected by syndromic (*p* < 0,001) or metabolic diseases (*p* < 0,001), children with a true microcephaly (*p* < 0,001), AED resistant nonlesional epilepsy patients (*p* = 0,030) and medical devises users (*p* < 0,001) were more likely to require at least one diagnostic procedure. No statistical significance was found for other conditions, nevertheless the trend was the same.

**Table 4:** Performances related to complex chronic conditions

	Number of diagnostic procedures				***P*** value^a^
Etiological diagnosis	0	1	≥2	Total	
Syndromic disorders	554 (29.3)	697 (36.9)	640 (33.8)	1891	< 0.001
Inherited metabolic diseases	89 (22.5)	194 (49.1)	112 (28.4)	395	< 0.001
Neuromuscular diseases	56 (29.0)	61 (31.6)	76 (39.4)	193	0.122
Congenital skeletal condition	17 (30.4)	14 (25.0)	25 (44.6)	56	0.103
True microcephaly	4 (16.0)	21 (84.0)	0	25	< 0.001
Isolated CNS malformations	49 (25.3)	72 (37.1)	73 (37.6)	194	0.744
Epilepsy under AED	94 (21.5)	188 (43.0)	155 (35.5)	437	0.030
Medical devices users	54 (18.8)	95 (33.0)	139 (48.3)	288	< 0.001

AED anti-epilepsy drugs CSN: central nervous system

^a^ Chi-squared test

1889/3479 (54.3%) patients were discharged home while 1590/3479 (45.7%) required hospitalization. PedEDs demonstrated a higher hospitalization rate compared to DedEDs and FunEDs (*p* < 0,001).

## Discussion

CSHCN affected by chronic conditions and disability often require complex, multidisciplinary, long-term assistance involving not only a general pediatrician but also the assistance from other specialists. To date, the number of CSHCN is increasing, and so are their healthcare needs. Due to their fragile underlying condition, CSHCN are vulnerable to health fluctuations and may often require emergency care.

Berry et al., assessed the access to the ED of children with chronic conditions with a retrospective analysis [14]. From the 1,850,027 studied cases, children suffering from sickle cell anaemia, epilepsy and asthma had the highest rates of visits to the ED. Even though the study referred to chronic conditions, and not specifically to children with special health care needs and disability [14].

In a retrospective study of 77,748 children visited at a PedED, 20% of the visits were of children with chronic conditions who consequently seemed to require a longer recovery time before being discharged. Moreover, the admission rates and the PedEDs recovery time increased according to their medical complexity [15].

Another study showed that, in spite of the patient’s medical complexity, the vast majority of children and young adults with multiple complex chronic conditions were visited in general EDs, and not in PedEDs. Moreover, the authors underlined that the admission rate and the total number of hospitalized patients did not differ between pediatric and general EDs [16].

Recently, Coller et al. conducted a retrospective study analyzing 271,806 visits in 37 American EDs. The study showed that 1 out of 4 children with a chronic condition visited at a children’s hospital ED was admitted to the hospital. A substantial variation among hospitalization rates of these children was noted across the EDs. EDs with the highest admission rates admitted more than 1 out of 3 children. History of prior hospitalizations, ED arrival overnight and clinical complexity were associated to higher rates of hospitalization [17].

The current study reflects the actual Italian EDs distribution, with FunEDs being the most represented. Data suggest that CSHCN generally refer to PedEDs instead of other less specialized EDs. The authors consider this concept of much relevance, because not every Italian region is provided with a PedEDs [13]. In fact, most EDs that visit children are not PedEDs. A possible explanation to this observation may be attributable to the fact that families with CSHCN would rather refer to a PedED where their child is already followed up on a regular basis, even if the hospital is far away from their home, than going to the nearest ED.

However, data showed that a great proportion of ED visits (44.2%) were registered during the night-time and/or at the weekends, independently from the type of ED. This seems to be in contrast to with what previously stated, suggesting that probably families who refer to a PedED might actually live closer to the children’s specialized hospital.

Considering the reason of access and the types of EDs, all etiological categories were admitted at both PedEDs and FunEDs. At the DedEDs, patients were mostly syndromic. Possible explanation to this phenomenon may be due to the number of syndromic patients, representing the most numerous and also the most complex category of them all. As a matter of fact, children affected by milder phenotypes might refer to the nearest ED, even if less specialized, where they are probably already known as patients.

Users of medical devices referred generally to PedEDs, emphasizing that certain devices could be managed mainly by specialized centers.

Regarding general pediatric population, the great majority of ED access (> 85%) were non-urgent, classified as white/green triage codes [18]. A greater number (51.4%) of urgent codes (red/ yellow codes) were evidenced. Nonetheless, green/white codes represented only 48.6% of the cohort. CSHCN seem to be at a higher risk of urgent access, although it is difficult to distinguish if it is due to the severity of the disease or to a more cautionary approach during the triage. For this kind of patients, indeed, there is a natural tendency to overtriage. This is a common behavior of the triage staff and a general rule for the triage system. Therefore, the triage code may not be representative of the actual severity of the condition, but rather of the priority of these particular patients for attention in the ED flow.

According to the distribution of yellow/red codes and the type of ED, it seems evident that patients with more severe or acute problems rather refer to a PedED, while non-urgent codes refer more frequently to FunEDs or DedEDs.

Since patient’s residence was not taken in consideration, the risk undertaken by parents/caregivers in order to reach to a more specialized Hospital was not evaluated.

Merrill et al., in 2007 presented a statistical summary on the ED admissions in children and adolescents without chronic conditions. Respiratory disorders accounted for 28% of all the ED admissions. Secondarily, injuries and gastrointestinal disorders represented the second most common causes of ED access, accounting for 17 and 14% respectively [19].

Regarding CSHCN, the present study confirmed that the most common reason for admission to the ED were respiratory symptoms (26.6%) and gastrointestinal problems (21.3%), similarly to what described for children without chronic diseases [18]. On the contrary, trauma represented a rare (3.6%) reason for ED admission in CSHCN, possibly due to the limitation of movement of the cohort. CSHCN are generally affected by neurological problems, hence explaining the higher frequency of acute neurological complications as a reason for ED access (18.2%) in comparison to non-chronic children (7%) [19]. Another specific reason for access to the ED is represented by device malfunction (3.9% of total accesses).

Analyzing the reasons of access per type of ED, it was interestingly noticed that patients with neurological problems and device malfunction referred mostly to PedEDs.

From this remarkable observation, it is possible to state that in case of complex medical conditions more specialized EDs are preferred. Additionally, data suggest that also concerning device malfunction which every ED should be able to handle, families do not consider the other EDs (FunEDs and DedEDs) reliable enough. Therefore, it would be desirable for all Italian EDs to improve their knowledge on medical device management in order to offer more efficient care to families.

The higher proportion of respiratory, osteoarticular problems and trauma in DedEDs is probably due to closer and easier access to these EDs. About half of patients assessed at the ED required hospitalization. The hospitalization rate appears to be higher in comparison to general pediatric population, with an estimation of 7–8% in Italian EDs [18, 20]. This evidence could be explained by both the severity and complexity of these children and/or by the need of a more careful attitude towards fragile children. The different percentage of hospitalization rate between PedEDs and FunEDs can be easily explained by the level of severity of the access, as evidenced by the triage severity codes.

As expected, a higher requirement for medical work up and procedures in these children was observed. Accordingly, a great number of them received intravenous fluids treatment, pharmacologic and oxygen administration. Moreover, 2.5% of the patients from the cohort underwent invasive procedures. No data regarding the medical procedures conducted in general pediatric population at the ED is available.

Looking at the different etiological categories a trend was demonstrated: syndromic, metabolic and epileptic patients are most likely to undergo more than one therapeutic procedure.

Children who attended to a PedED were more likely to undergo to a higher number of medical procedures in comparison to other EDs independently from the outcome (hospitalization or discharge). Probably, this phenomenon could be explained by the different triage code severity but also by the skilled attitude in facing patients with this kind of problems at the PedEDs.

The study presents some clear limitations reported as follows. On the one hand, it is impossible to tell if all fragile patients evaluated in the different EDs during the defined period were actually included in the study. On the other hand, due to the higher interest of PedEDs towards the purpose of the study, possible selection bias cannot be certainly abolished. Moreover, lack of information regarding patient’s residence and address makes hard to tell whether the chosen ED by parents/caregivers is related to the logistics (location) or to the Hospital facilities. The lack of the total number of pediatric visits in the various EDs during the observation period represents another limitation, since without this information it was not possible to calculate the rate of CSHCN visits among all pediatric ED visits. In spite of these limitations, this survey provides a preliminary description of this phenomenon and could represent a good starting point for further and more specific studies on the matter.

## Conclusion

This is the first report of access to Italian EDs in CSHCN. According to the study analysis, the access of this kind of patients are not infrequent independently from the type of ED. As expected, clinical severity, number of medical workup and the hospitalization rates are higher in respect to general pediatric population. Therefore, it is crucial for pediatricians working in an ED to increase their general knowledge and expertise on the management of these kind of patients.

## Data Availability

The raw data supporting the conclusions of this manuscript will be made available by the authors without undue reservation to any qualified researcher.

## Abbreviations

CSHCN: Children with special health care needs
ED: Emergency department
PedED: Pediatric ED, FunED Funtional ED
DedED: Dedicated ED
CPR: Cardiopulmonary resuscitation
AED: Anti-epilepsy drugs
CSN: Central nervous system
